# Knowledge and Attitude of Dental Practitioners Related to Disinfection during the COVID-19 Pandemic

**DOI:** 10.3390/healthcare8030232

**Published:** 2020-07-25

**Authors:** Shaur Sarfaraz, Juzer Shabbir, Muhammad Adeel Mudasser, Zohaib Khurshid, Ahmed Adel A. Al-Quraini, Maria Shakoor Abbasi, Jithendra Ratnayake, Muhammad Sohail Zafar

**Affiliations:** 1 Institute of Medical Education, Jinnah Sindh Medical University, Karachi 75510, Pakistan; shaur.sarfaraz@jsmu.edu.pk; 2 Operative Dentistry Department, Liaquat College of Medicine and Dentistry, Karachi 75290, Pakistan; dr.juzer.shabbir@gmail.com; 3 Dr. Ishrat-Ul-Ebad Khan Institute of Oral Health Sciences, Dow University of Health Sciences (DUHS), Karachi 74200, Pakistan; adeel.mudasser@duhs.edu.pk; 4 Department of Prosthodontics and Dental Implantology, College of Dentistry, King Faisal University, Al-Ahsa 31982, Saudi Arabia; 5 Department of Dental Hospital, College of Dentistry, King Faisal University, Al-Ahsa 319825, Saudi Arabia; aalquraini@kfu.edu.sa; 6 Department of Prosthodontics, Altamash Institute of Dental Medicine, Karachi 75500, Pakistan; maria_shakoor@hotmail.com; 7 Department of Oral Sciences, Faculty of Dentistry, University of Otago, Dunedin 9016, New Zealand; jithendra.ratnayake@otago.ac.nz; 8 Department of Restorative Dentistry, College of Dentistry, Taibah University, Al-Madina Al-Munawwarah 41311, Saudi Arabia; mzafar@taibahu.edu.sa; 9 Department of Dental Materials, Islamic International Dental College, Riphah International University, Islamabad 44000, Pakistan

**Keywords:** disinfection, SAR-CoV-2, surfaces, hygiene, COVID-19, dentistry, mouthwash

## Abstract

The world is currently facing a pandemic crisis due to a novel coronavirus. For this purpose, acquiring updated knowledge regarding prevention and disinfection during the current pandemic is necessary for every dental practitioner. In our study, we aimed to evaluate globally the level of knowledge and the attitude of dental practitioners related to disinfection. A total of 385 participants out of 401 participants from 23 different countries across the world were included in the final analysis after the exclusion of incomplete responses. The majority of the dentists who responded were females (53.8%) and were practicing at private health institutes (36.4%). The mean knowledge score of the participants was estimated to be 4.19 ± 1.88 out of 12, reflecting insufficient knowledge, and the mean attitude score of the participants was estimated to be 12.24 ± 3.23 out of 15, which shows a positive attitude toward disinfection practices during coronavirus 2019 (COVID-19). Thus, the current study indicated a lack of knowledge in fundamental aspects of disinfection protocols with a significant and positive attitude from dental health professionals toward disinfection regarding the coronavirus 2019 (COVID-19) pandemic.

## 1. Introduction

The world is currently in a hold of an insidious virus that is wreaking havoc everywhere. This virus is considered as a new strain of the coronavirus family. It was first termed as 2019 nCoV (2019 novel coronavirus) by the Chinese scientists, and lately coronavirus 2019 (COVID-19) by the World Health Organization (WHO) [1]. The virus was found to be genetically similar to previous bat-originated severe acute respiratory syndrome (SARS)-like corona-viruses [2]. However, the origin of COVID-19 is still unclear [2,3]. The illness caused by this virus ranges from asymptomatic or mild symptoms to more severe clinical manifestations, including severe respiratory distress requiring mechanical ventilation [4]. According to the World Health Organization (WHO), more than 5.8 million COVID-19 confirmed cases have already been reported globally (https://covid19.who.int/).

The International Committee on the Taxonomy of Viruses named this virus severe acute respiratory syndrome-coronavirus-2 (SARS-CoV-2), and it has been shown to spread from person to person via respiratory droplets through coughing, sneezing, or even talking; by physical contact with an infected person; or by touching a contaminated surface [5]. The SARS-CoV-2 is stable at 4 °C for up to 14 days [6]. When the temperature is increased to 70 °C, its infectivity remains only for 5 minutes. Similarly, coronaviruses have been shown to persist on inanimate surfaces, such as metal, plastic, or glass for up to a period of 9 days or until disinfected [7]. Currently, no treatment for COVID-19 is available. Few potential therapies are in the trial phase leaving mainly supportive and preventive strategies against this disease [5]. The rapid testing of this disease is possible through blood, saliva, and nasopharyngeal swabs [8]. With the help of rapid point-of-care (POC) technology, transmission can be controllable [9].

Various organizations and groups have suggested their own preventive and disinfection strategies against the transmission of SARS-CoV-2 to doctors and health care workers. These strategies are crucial, especially for dental practitioners, presumably due to their increased potential to become infected due to the very nature of the field itself. These strategies include various recommendations for minimizing exposure, disinfecting the dental care practice, and managing waste. The dental practitioners and practices have the potential to spread the disease to the population unknowingly, if the precautions are not being strictly followed. A well-documented global survey reported on the fear and dental practice modification, on the need for following international guidelines from dental regulatory bodies in the emergency treatment, and also on the educational programs mandatory for reducing the fear and anxiety of this crisis in routine procedures [10].

For this purpose, acquiring updated knowledge regarding prevention and disinfection during the current pandemic is necessary for every dental practitioner. Our study aimed to evaluate the level of knowledge and attitude of the dental practitioners related to disinfection globally.

## 2. Materials and Methods

The current study was cross-sectional, conducted from 14th May 2020 to 20th May 2020 through an online survey. A minimum sample size of 355 was estimated using the Open epi website (www.openepi.com) by taking statistics as follows: 36.1% for the correct response for the incubation period by dentists, the margin of error as 5%, and a 95% confidence level [11]. To address the potential of drop-out, the sample size was increased by 20%. Hence, the final sample size was found to be 426. The ethical approval of the study was obtained from the ethics committee, Altamash Institute of Dental Medicine, Karachi. An informed consent was taken from all the participants before the start of the survey. The eligibility criteria consisted of qualified male or female dental healthcare professionals globally, aged 25 years or more, including dental practitioners and clinical post-graduate students who understood the content of the survey and agreed to participate in the study. The link to the online questionnaire was provided to the participants through various social media platforms and emails. The participants were instructed to complete the questionnaire by clicking the link or scanning the quick response (QR) code.

The online questionnaire consisted of 17 items and a brief introduction, including the objective, procedures, declarations of anonymity and confidentiality, and notes for filling the questionnaire. The questionnaire had three sections. Section one consisted of questions related to personal information or demographic data (age, gender, years of practice, and present working place).

Section two consisted of questions pertaining to their knowledge of disinfection against COVID-19. This section contained ten questions (1–8 were single choice questions and the last two questions, 9 and 10, were multiple choice questions) regarding the stability of SARS-CoV-2 on different surfaces commonly found in a dental clinic, such as tissue and printing paper, glass, plastic, stainless steel, and inanimate surfaces; the choice and effectiveness of mouth rinses; hand sanitizers of choice; and information regarding disinfectants used for the cleaning of various surfaces against SARS-CoV-2. The answers for these questions were composed of “correct”, “incorrect”, and “don’t know” options. The correct answer was assigned 1 point, whereas incorrect or unknown answers were assigned 0 points. The total score for this section ranged from 0 to 12, with a higher score indicating a better knowledge of disinfection against COVID-19. The score of the participants was further divided into two categories with a cut-off value (on the basis of the median value) of 7 (60%). The scores of ≥7 were characterized as “sufficient knowledge” and <7 were labelled as “insufficient knowledge”.

Section three consisted of statements (to be agreed or disagreed) related to their attitude toward disinfection against COVID-19. This section contained three statements regarding the risk of COVID-19, the disinfection required, and disinfection guidelines for COVID-19. These statements were associated with a 5-point Likert scale (from “strongly disagree” to “agree strongly”) options. The overall score in this section was in the range of 3 to 15. The score of >6 indicated a positive (or satisfactory) attitude and ≤6 indicated a negative (or unsatisfactory) attitude related to disinfection. Previously, a study concluded the need for educational campaigns and patient identification for good practices regarding Middle East Respiratory Syndrome Corona Virus (MERS-COV) in Saudi Arabia [12]. Another study recently reported on the dental practice modification according to recent healthcare guidelines to combat the fear of COVID-19 pandemic. All these surveys shared a message of new educational learning needs for the betterment of dental practice [12]. The reliability of the questionnaire was checked using the pilot data of 30 participants, and the Cronbach’s alpha value was estimated as 0.73.

The data were analyzed using SPSS version 23 (IBM, Armonk, NY, USA). The mean and standard deviation (SD) or median and interquartile range (IQR) were computed for the numeric variables, whereas the frequency and percentage were computed for the categorical/nominal variables. The chi-square or Fisher exact tests were applied to assess the association between the effect modifiers and outcome variables, i.e., knowledge and attitude level. *p* ≤ 0.05 was taken as statistically significant.

## 3. Results

Out of 426 participants, 401 responded (response rate = 94.1%). Three hundred and eighty-five participants were included in the final analyses after the exclusion of incomplete responses. The participants’ ages ranged from 25 to 73 (mean = 35.18) years. The median years of experience were estimated at 7 years. The majority of the dentists were females (53.8%) and were practicing at private health institutes (36.4%). The majority of the study participants lived in Pakistan (43.9%), followed by Saudi Arabia (20%), New Zealand (9.4%), Australia, the UK, the US, and Brazil (4.2%). Fewer responses were received from other countries, such as Thailand (6), the UAE (5), China (4), Japan (4), Hungary (4), and India (3). Two responses were received from Canada, Nepal, and Israel. Only one response was received from Switzerland, Bangladesh, Malaysia, Poland, Belgium, Lebanon, and Panama, each (Table 1).

The assessment of the knowledge of participants regarding disinfection against SAR-CoV-2 showed that 33.8% of the participants were aware of the protocols for cleaning visibly soiled hands with the use of soap and water for 20 s and then disinfection with alcohol-based hand rub (ABHR). Similarly, 43.6% of the participants were aware that the efficiency of a disinfectant against SAR-CoV-2 depends upon its contact time with the surface. Only 1.6% of the participants knew that the coronavirus could remain infectious on inanimate surfaces for up to 9 days. Moreover, 17.9% of participants were aware that SAR-CoV-2 could remain infectious on printing and tissue papers for up to 30 min. We found that 11.9% of the participants knew that SAR-CoV-2 could remain infectious on stainless steel and plastic for up to 7 days (Table 2).

The majority of the participants (66.8%) were aware that disinfectants containing 1000 mg/L chlorine should be used against coronavirus for the disinfection of floors, walls, and dental arbitrary/operatory. We found that 46% of the participants knew that 75% to 80% alcohol-based hand sanitizer is effective against SAR-CoV-2. Similarly, 52.2% of the participants knew that sodium hypochlorite is the recommended disinfectant against SAR-CoV-2 for the disinfection of waste before disposal. Of the participants, 22.6% and 36.9% were aware that 0.23% to 7% povidine-iodine (PVP I) and 1.5% hydrogen peroxide are recommended as a pre-procedural mouth rinse to reduce the viral load. Additionally, 41% and 29.6% of the participants knew that the surface disinfectants effective against coronavirus include 0.1% sodium hypochlorite, 0.5% hydrogen peroxide, and 62%–71% ethanol (Table 2). The average knowledge score of participants was estimated to be 4.19 ± 1.88.

The assessment of attitudes toward disinfection against coronavirus showed that 54.5% of the participants strongly agreed that there is a high risk of contracting the SARS-COV-2 in dental practice. We found that 31.9% of the participants strongly agreed that following the disinfection guidelines will help reducing the risk of becoming infected with coronavirus. The majority of the participants (66.5%) strongly agreed that it is essential to disinfect the frequently touched surfaces in a dental clinic during the COVID-19 pandemic (Table 3).

**Figure 1:**
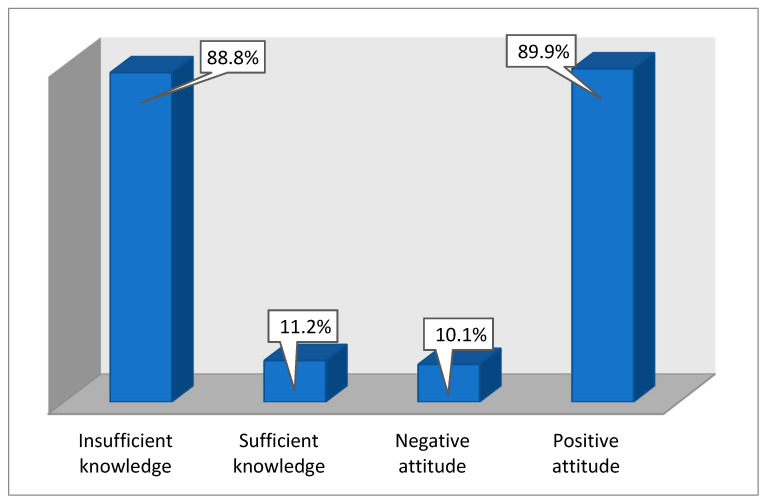
Frequency distribution of knowledge and attitude levels.

The average attitude score of the participants was estimated to be 12.24 ± 3.23. According to the criteria of scoring, the majority of the participants (88.8%) had inadequate knowledge regarding disinfection against SARS-CoV-2. Similarly, the majority of the participants (89.9%) showed a positive attitude toward disinfection against SARS-CoV-2 (89.9%) (Figure 1).

The knowledge level showed a statistically significant difference with respect to the years of practice and country of the dental practitioners (*p* < 0.05) (Table 4).

## 4. Discussion

In the current COVID 19 pandemic crisis, there are no approved antiviral agents, drugs, or vaccines available for protection against this deadly disease. However, the remaining effective mitigating strategy is to reduce the transmission of the virus (through droplets or close contact) [13]. Several guidelines and strategies have been suggested to prevent and control the disease at three levels: the case-related population, the general population, and the national level. These include the maintenance of hand hygiene, disinfection of surfaces, and adherence to basic cough etiquette [14,15]. Similarly, the National Health Commission of China (NHCC) issued disinfection protocols for the elderly and rural area populations that can be applied all over the world [13,14,15,16,17]. The World Health Organization (WHO) also suggested guidelines for the reduction of viral load through the cleaning and disinfection of surfaces and wastes with the help of disinfectants, such as 0.1% sodium hypochlorite, 0.5% hydrogen peroxide, or 62%–71% ethanol [18,19].

The results of the current study suggested that almost 50% of the dental health professionals failed to correctly indicate the surface disinfectant that was effective against COVID-19. However, the majority were aware of the use of 1000 mL chlorine-containing disinfectant for cleaning the walls, floors, and dental arbitrary/operatory. This might be related to the prevalent misconception (57%) among participants in our study that the effectiveness of disinfectant is not dependent on the contact time with the surfaces. Likewise, 50% of the participants thought that handwashing with only soap and water was adequate when hands were visibly soiled. On the contrary, based on the previous studies [20,21] related to Severe Acute Respiratory Syndrome Corona Virus (SARS-CoV) and Middle East Respiratory Syndrome Corona Virus (MERS-COV), the WHO recommended handwashing with soap and water for 20 seconds followed by alcohol-based hand rub (ABHR) for visibly soiled hands.

We also found that the participants lacked knowledge related to the stability of COVID-19 on different surfaces. Researchers reported that COVID-19 remained stable on inanimate surfaces up to 9 days; on tissue or printing papers for up to 3 h; wood and clothes for up to 2 days; smooth surfaces, like glass and banknotes, for 4 days; stainless steel, the inner surface of surgical masks, and plastic for 7 days; and on the outer layer of surgical masks for even more than 7 days [22,23]. Similarly, most of the participants thought that chlorhexidine is currently recommended as a pre-procedural mouth rinse. However, chlorhexidine has been discouraged from being used for the purpose of reducing the COVID-19 viral load. On the other hand, during the COVID-19 pandemic, 0.23% to 7% povidine-iodine (PVP-I) or 1.5% hydrogen peroxide-containing mouthwashes are recommended as a pre-procedural mouth rinse [24,25]. One study also suggested the potential use of mouthwashes containing cetylpyridinium chloride (CPC) 0.045% to 0.1% in the future against COVID-19. Recently, an in vitro study reported outcomes of 0.5%, 1%, and 1.5%, of PVP-I completely inactivating SARS-CoV-2 within 15 s of contact [12].

Table 4 in this study shows 75% to 100% insufficient knowledge with 83% to 100% positive attitude among different age, gender, years of practicing, and institutes (public/private/private practice) and countries responded. Significant statistical differences were found when the years of practicing were less than 10 years of experience. These dental health professionals had insufficient knowledge and all responded countries demonstrated a significant lack of knowledge regarding disinfection.

Although there is a nexus of different content regarding disinfection, this survey made it possible to collect the important information related to disinfection and reported the need for improving the knowledge of dental health professionals all over the world. The limitations of this study include that this is a cross-sectional study that can only prove association and not a cause–effect relationship and that the data was collected in a limited time period, keeping in mind the rapid effect this outbreak was having on the updated knowledge regarding disinfection and its guidelines. We accessed the knowledge and attitude of dental health practitioners but were not able to study the impact of dental management practices among dentists, and this may be assessed in future studies once they are allowed to practice, which was not applicable in our case. Furthermore, we did not receive responses from all countries that have been affected by the outbreak. Hence, the generalizability of the study is limited.

## 5. Conclusions

Despite exceptional guidelines regarding disinfection against SARS-CoV-2, the current study indicated a lack of knowledge in dentists about fundamental aspects of disinfection protocols. We also found a significant and positive attitude of dental health professionals toward disinfection regarding the COVID-19 pandemic. This indicates that our health professionals have good intentions to practice appropriately but do not have adequate knowledge to implement disinfection guidelines specifically against COVID 19. To spread awareness regarding disinfection control, there is a need to begin online sessions for health professions so they can upgrade their knowledge by understanding the recent guidelines to stay safe and protect others from becoming infected; this will help to reduce the spread of COVID-19.

## Figures and Tables

**Table 1:** Baseline information of study participants.

Sample Characteristics	Values (*n* = 385)
Age ^1^	35.18 ± 9.68
Years of practicing ^2^	7 (3–15)
Gender	
*Male*	178 (46.2%)
*Female*	207 (53.8%)
Health sector	
*Private*	135 (35.1%)
*Public*	140 (36.4%)
*Private practice*	110 (28.6%)
Country	
*Pakistan*	169 (43.9%)
*Saudi Arab*	77 (20%)
*New Zealand*	36 (9.4%)
*Australia*	16 (4.2%)
*US*	16 (4.2%)
*UK*	16 (4.2%)
*Brazil*	16 (4.2%)
*Other countries*	39 (10.1%)

^1^ Mean ± SD; ^2^ Median (Interquartile range, IQR).

**Table 2:** Knowledge of the participants regarding disinfection against severe acute respiratory syndrome-coronavirus-2 (SARS-CoV-2) and coronavirus 2019 (COVID-19).

Item	Responses	*n* (385)	%
1. It is recommended to clean visibly soiled hands with the use of:	Soap and water for 20 s	209	54.3
	70–85% Alcohol-based hand rub (ABHR)	43	11.2
	Soap and water for 20 seconds and then alcohol-based hand rub	130	33.8
	Don’t Know	3	8
2. The efficiency of a disinfectant against coronavirus depends on the:	Composition of a disinfectant	183	47.5
	Use of more than one disinfectant at a time	12	3.1
	Contact time of disinfectant on the surface	168	43.6
	Don’t know	22	5.7
3. Coronavirus can remain infectious on inanimate surfaces for:	3 days	224	58.2
	6 days	47	12.2
	9 days	64	16.6
	Don’t know	50	13.0
4. SARS-COV-2 can remain infectious on printing papers and tissue paper for:	2 h	38	9.9
	3 h	69	17.9
	4 h	122	31.7
	Don’t know	156	40.5
5. SARS-COV-2 can remain infectious on stainless steel and plastic for:	1 day	102	26.5
	7 days	46	11.9
	14 days	173	44.9
	Don’t know	64	16.6
6. The disinfectants used against coronavirus to disinfect floors, walls, and dental arbitrary/operatory should contain:	1000 mg/L Chlorine	257	66.8
	2 mg/L Ozone	7	1.8
	6 mg/L Peracetic Acid	24	6.2
	Don’t know	97	25.2
7. Effective hand sanitizer against coronavirus:	60% to 70% Alcohol-based hand sanitizer	182	47.3
	75% to 80% Alcohol-based hand sanitizer	177	46.0
	Alcohol-free hand sanitizer	6	1.6
	Don’t know	20	5.2
8. The recommended disinfectant used against coronavirus to disinfect waste before disposal include:	Hydrogen peroxide	71	18.4
	Chloroxylenol (Dettol)	61	15.8
	Sodium hypochlorite (bleach)	201	52.2
	Don’t know	52	13.5
9. The recommended mouthwashes as a pre-procedural rinse to reduce viral load include:	0.045% to 0.1% Cetylpyridinium chloride (CPC)	45	11.7
	0.23% to 7% Povidine-Iodine (PVP I)	87	22.6
	0.5% Chlorhexidine	207	53.8
	1.5% Hydrogen peroxide	142	36.9
	Don’t Know	36	9.4
10. Surface disinfectants effective against coronavirus include:	0.1% Sodium hypo-chloride, 0.5% hydrogen peroxide within 1 min	158	41.0
	0.1% Sodium hypo-chloride and 42–61% ethanol within 20 s	148	38.4
	62–71% ethanol within 1 min	114	29.6
	0.02% Chlorhexidine di-gluconate within 2 min	40	10.4
	Don’t Know	86	22.3

**Table 3:** Attitude toward disinfection against SARS-CoV-2.

Items	Strongly Disagree	Disagree	Neutral	Agree	Strongly Agree
1. There is a high risk in a dental practice for becoming infected by SAR-CoV-2	58 (15.1%)	8 (2.1%)	23 (6%)	86 (22.3%)	210 (54.5%)
2. Following the disinfection guidelines will help in reducing the risk of becoming infected by SARS-CoV-2	26 (6.8%)	7 (1.8%)	44 (11.4%)	185 (48.1%)	123 (31.9%)
3. It is important to disinfect the frequently touched surfaces in a dental clinic during the COVID-19 pandemic	42 (10.9%)	2 (0.5%)	16 (4.2%)	69 (17.9%)	256 (66.5%)

**Table 4:** Comparison of knowledge and attitude with the baseline characteristics of dentists.

Variables	Knowledge		*p*-Value	Attitude		*p*-Value
	Insufficient	Sufficient		Negative	Positive	
**Age groups**						
25–50 years	314 (89.2%)	38 (10.8%)	0.395	37 (10.5%)	315 (89.5%)	0.557
51–75 years	28 (84.8%)	5 (15.2%)		2 (6.1%)	31 (93.9%)	
**Gender**						
Male	161 (90.4%)	17 (9.6%)	0.418	20 (11.2%)	158 (88.8%)	0.612
Female	181 (87.4%)	26 (12.6%)		19 (9.2%)	188 (90.8%)	
**Years of practicing**						
≤10 years	229 (90.9%)	23 (9.1%)	0.023	28 (11.1%)	224 (88.9%)	0.383
11–20 years	71 (86.6%)	11 (13.4%)		8 (9.8%)	74 (90.2%)	
21–30 years	33 (89.2%)	4 (10.8%)		2 (5.4%)	35 (94.6%)	
31–40 years	5 (55.6%)	4 (44.4%)		0	9 (100%)	
41–50 years	4 (80%)	1 (20%)		1 (20%)	4 (80%)	
**Institute type**						
Private	122 (90.4%)	13 (9.6%)	0.121	11 (8.1%)	124 (91.9%)	0.46
Public	128 (91.4%)	12 (8.6%)		16 (11.4%)	124 (88.6%)	
Private practice	92 (83.6%)	18 (16.4%)		12 (10.9%)	98 (89.1%)	
**Country**						
Pakistan	153 (90.5%)	16 (9.5%)	0.023	16 (9.5%)	153 (90.5%)	0.308
Saudi Arabia	73 (94.8%)	4 (5.2%)		13 (16.9%)	64 (83.1%)	
New Zealand	31 (86.1%)	5 (13.9%)		2 (5.6%)	34 (94.4%)	
Australia	12 (75%)	4 (25%)		2 (12.5%)	14 (87.5%)	
US	14 (87.5%)	2 (12.5%)		1 (6.3%)	15 (93.8%)	
UK	16 (100%)	0		2 (12.5%)	14 (87.5%)	
Brazil	12 (75%)	4 (25%)		1 (6.3%)	15 (93.8%)	
Other countries	31 (79.5%)	8 (20.5%)		2 (5.1%)	37 (94.9%)	
